# The New Physiological Buildings at Cardiff

**Published:** 1915-08-28

**Authors:** 


### THE NEW PHYSIOLOGICAL BUILDINGS AT CARDIFF.

### Sir William Osler's Wise Counsel.

In our issue of last week a *résumé* was given of Sir William James Thomas' speech on the occasion of laying the foundation stone of the new Physiological Buildings at Cardiff. If space permitted, extracts from other of the excellent speeches then delivered would prove interesting reading, but in any event Sir William Osler's remarks should be noted. Sir William said they had in Cardiff a first-class, up-to-date, modern hospital, into which they could send their medical students with a full knowledge that they would receive a thorough scientific and practical training. They had in connection with the hospital a good pathological department in charge of a whole-time professor—a department that was doing first-class work, and thoroughly equipped and established. That was as far as they had gone in this great enterprise of the medical school. Next they came to what was in sight. In the first place, the completion of the physiological buildings would give them the satisfaction of knowing that they had in this medical school as good a physiological department as there was anywhere in the kingdom or anywhere in the world. Secondly, they would have an anatomical laboratory, a splendid public health and pharmacological laboratory, and a central library, which were conditions which the donor (Sir William James Thomas) had been safe in making. If they could see these completed they would have all the equipment for a great medical school. He had spoken of what had been done, what was in sight, and the further difficulty was what remained. They had got to face the problem of organising their departments in the hospital on university lines. They must be units in the university scheme, and they must devise some means by which the university and the hospital would work in co-operation in furthering these great necessities in a great medical school. Then there was the difficulty as to the relation of the medical school to the University of Wales and the university colleges. They had to recognise that they could not in a small Principality like Wales have these big universities. They could have three big colleges, but only one university, and in a small and comparatively poor country they must not duplicate their departments. Another question came up, and it was a very important one, as to the status and mode of appointment of the professors. He commended the system of appointing professors which was adopted at Oxford and Cambridge, where a faculty of experts from all over the kingdom met and chose the best man. If they started with the idea that they were going to have a local university with only Welsh brains, he would be sorry for the donor that day. They must in wisdom go into the market and buy the best brains of the country, because the best brains of the country were not one whit too good for Wales. Sir William considered the prospect of Treasury help favourable, as the Treasury would help those who helped themselves, and that day was an earnest of what Wales is capable of doing in that direction.

